# Role of surgical hyoid bone repositioning in modifying upper airway collapsibility

**DOI:** 10.3389/fphys.2022.1089606

**Published:** 2022-12-13

**Authors:** Corine J. Samaha, Hiba J. Tannous, Diane Salman, Joseph G. Ghafari, Jason Amatoury

**Affiliations:** ^1^ Sleep and Upper Airway Research Group (SUARG), American University of Beirut, Beirut, Lebanon; ^2^ Division of Orthodontics and Dentofacial Orthopedics, American University of Beirut Medical Center, Beirut, Lebanon; ^3^ Biomedical Engineering Program, Maroun Semaan Faculty of Engineering and Architecture (MSFEA), American University of Beirut, Beirut, Lebanon; ^4^ Department of Orthodontics, University of Pennsylvania, Philadelphia, PA, United States

**Keywords:** upper airway surgery, upper airway patency, pharynx, closing pressure, rabbit model, obstructive sleep apnea, OSA, sleep-disordered breathing

## Abstract

**Background:** Surgical hyoid bone repositioning procedures are being performed to treat obstructive sleep apnea (OSA), though outcomes are highly variable. This is likely due to lack of knowledge regarding the precise influence of hyoid bone position on upper airway patency. The aim of this study is to determine the effect of surgical hyoid bone repositioning on upper airway collapsibility.

**Methods:** Seven anaesthetized, male, New Zealand White rabbits were positioned supine with head/neck position controlled. The rabbit’s upper airway was surgically isolated and hyoid bone exposed to allow manipulation of its position using a custom-made device. A sealed facemask was fitted over the rabbit’s snout, and mask/upper airway pressures were monitored. Collapsibility was quantified using upper airway closing pressure (Pclose). The hyoid bone was repositioned within the mid-sagittal plane from 0 to 5 mm (1 mm increments) in anterior, cranial, caudal, anterior-cranial (45°) and anterior-caudal (45°) directions.

**Results:** Anterior displacement of the hyoid bone resulted in the greatest decrease in Pclose amongst all directions (*p* = 0.002). Pclose decreased progressively with each increment of anterior hyoid bone displacement, and down by −4.0 ± 1.3 cmH_2_O at 5 mm. Cranial and caudal hyoid bone displacement did not alter Pclose (*p* > 0.35). Anterior-cranial and anterior-caudal hyoid bone displacements decreased Pclose significantly (*p* < 0.004) and at similar magnitudes to the anterior direction (*p* > 0.68).

**Conclusion:** Changes in upper airway collapsibility following hyoid bone repositioning are both direction and magnitude dependent. Anterior-based repositioning directions have the greatest impact on reducing upper airway collapsibility, with no effect on collapsibility by cranial and caudal directions. Findings may have implications for guiding and improving the outcomes of surgical hyoid interventions for the treatment of OSA.

## 1 Introduction

The hyoid is a small and uniquely freely suspended bone located at the base of the tongue. The hyoid bone serves as an insertion point to several upper airway dilator muscles. As such, an abnormally positioned hyoid bone may affect upper airway tissue mechanical properties as well as the mechanical effectiveness of pharyngeal muscles in maintaining upper airway patency. A more inferiorly positioned hyoid bone is the most consistently observed craniofacial anatomical trait in individuals with obstructive sleep apnea (OSA) (Sforza et al., 2000; Chi et al., 2011; Genta et al., 2014), a disorder characterized by repetitive partial or complete collapse of the upper airway that can lead to serious health consequences (Lal et al., 2012; Kimoff, 2016; Veasey and Rosen, 2019). A more inferior position of the hyoid bone has also been associated with more severe OSA, as characterized by the apnea-hypopnea index (AHI) (Costa e Sousa and dos Santos Gil, 2013; Stipa et al., 2019), and with increased upper airway collapsibility (Sforza et al., 2000; Genta et al., 2014).

Surgical procedures on the hyoid bone have been advocated to treat OSA. These include hyomandibular suspension, which involves displacing the hyoid anterior-cranially by anchoring it to the inferior border of the mandible (Neruntarat, 2003), and hyothyroidopexy, through which the hyoid is repositioned more caudally by way of attachment to the thyroid cartilage (Riley et al., 1994). The reduction of AHI with both procedures are unpredictable and variable amongst patients, with success rates ranging from 17%–78% (Neruntarat, 2003; Bowden et al., 2005; den Herder et al., 2005; Kezirian and Goldberg, 2006). Currently, hyoid repositioning surgeries are being performed without individual prescription for magnitude and direction of hyoid displacement, or appreciation of the effect that the new hyoid position will have on upper airway function (Riley et al., 1986; Schmitz et al., 1996). Anterior movement of the hyoid has been shown to stabilize the hypopharynx and reduce the tendency for collapse under negative intraluminal pressures in animal and human cadaver studies (Van de Graaff et al., 1984; den Herder et al., 2005; Rosenbluth et al., 2012). However, little attention has been given to quantify the effect of different hyoid displacement magnitudes and directions on upper airway outcomes.

The aim of this study is to investigate the influence of surgical hyoid repositioning on upper airway collapsibility using an anaesthetized rabbit model. The rabbit is an ideal model to study the upper airway and hyoid given its similar structure to humans, whereby the rabbit has a freely suspended hyoid (compared with a fixed bone in most non-primates) (Amatoury et al., 2014; Amatoury et al., 2015), making it the ideal model for investigation.

## 2 Materials and methods

### 2.1 Subjects

Studies were performed in seven adult male New Zealand White rabbits [weight = 3.14 ± 0.39 kg (mean ± SD)]. The protocol was approved by the American University of Beirut Institutional Animal Care and Use Committee (#19-08-544).

### 2.2 Experimental setup

Anesthesia was initially induced with an intramuscular injection of ketamine (35 mg/kg) and xylazine (5 mg/kg) and then maintained with a continuous intravenous infusion (*via* an ear vein) of ketamine (15 mg/kg/hr) and xylazine (4.5 mg/kg/hr) at an infusion rate of 0.025 ml/min/kg. The rabbits were maintained in a physiologically stable state throughout the experiment, as determined by monitoring heart and respiratory rates. Animals were euthanized at the completion of each study with an anesthetic overdose.

**FIGURE 1:**
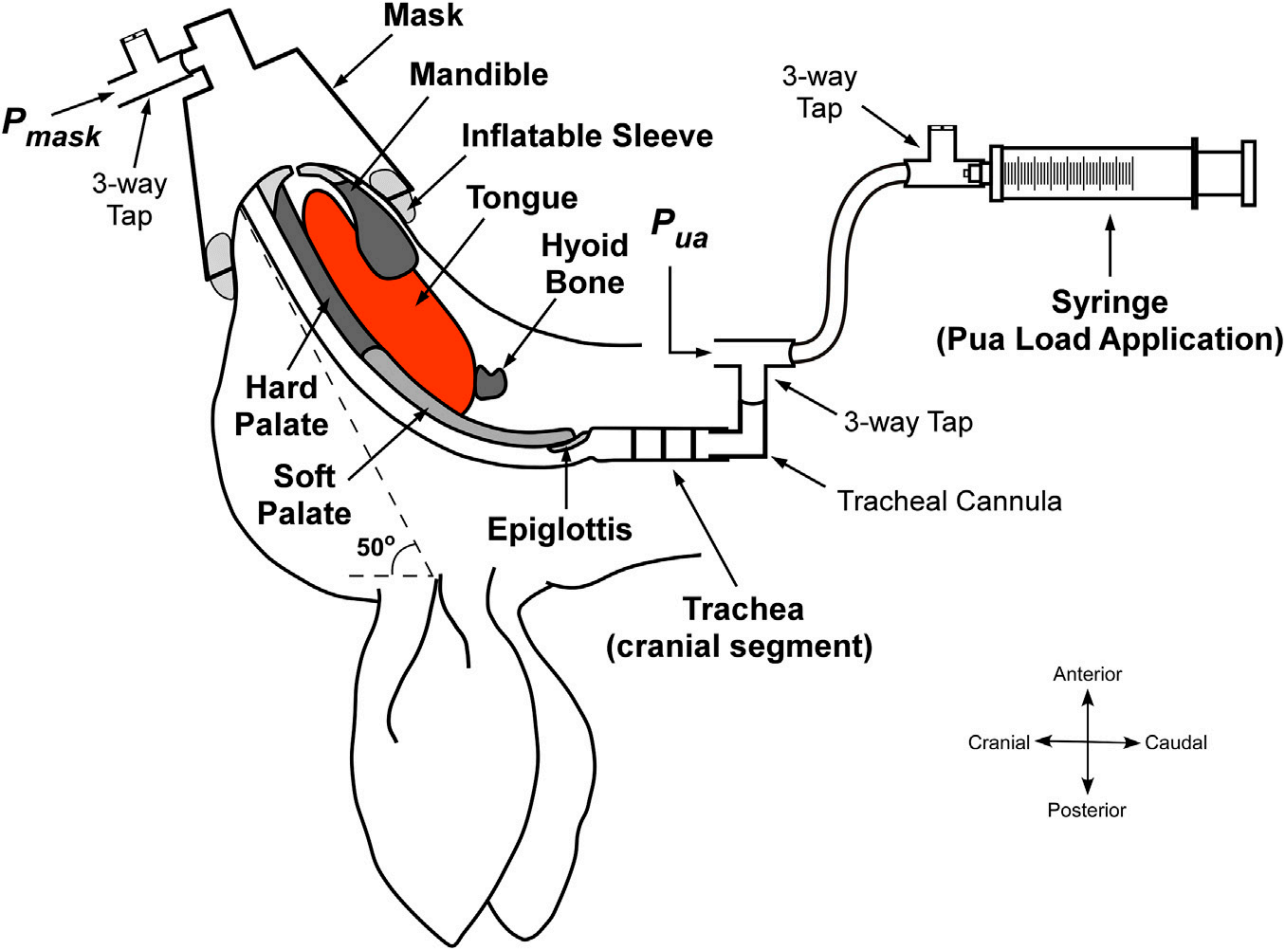
Schematic of the experimental setup with upper airway negative pressure application. The upper airway was isolated at the level of the trachea. A syringe is connected to the cranial tracheal segment and pulled to create a negative pressure in the upper airway, which is detected at the level of the mask. Pmask, mask pressure. Pua, pressure at the cranial end of the trachea. Adapted and modified from Amatoury, 2012 (Amatoury, 2012).

The animals were placed in a supine position with head/neck position controlled such that a line drawn from the tragus to the external nares was at 50° to the horizontal (Figure 1). The trachea and hyoid were surgically exposed *via* neck incision and blunt dissection. Hyoid muscle connections and associated nerves were carefully preserved during this step. The trachea was then severed between the third and fourth cartilaginous rings. Cranial and caudal tracheal segments were cannulated separately using custom-prepared L-shaped tubes. The rabbits were able to breathe spontaneously through the caudal trachea. The cranial tracheal segment was reextended to its pre-transection end-expiratory position. Additional connective tissue overlying the hyoid was carefully removed to further expose the hyoid in preparation for attachment of the hyoid displacement device (see below).

To enable upper airway collapsibility measurement, a 20 ml syringe was attached to the cranial tracheal cannula *via* a 3-way tap and 100 cm volume extension for negative upper airway application, and a differential pressure transducer (Validyne DP45–32; Validyne Engineering, Northridge, CA) to measure upper airway pressure (Pua). A small modified conical animal anesthetic mask (GaleMed VM-2, GaleMed, Taiwan) was fitted to the rabbit’s snout to achieve a closed upper airway system and for the measurement of mask pressure (Pmask; Validyne DP45–32). To secure an airtight seal around the rabbit’s snout, an inflatable rubber sleeve was incorporated around the internal base of the mask (Figure 1). Before fitting the mask, petroleum jelly (Vaseline) was also applied to the rabbit’s snout to further help minimize leakage.

To ensure that the rabbit was in a stable anesthetized state throughout the experiment, and later to confirm euthanasia, basic vital signals were monitored with electrocardiogram (ECG) as well as tracheal pressure, which was measured from the caudal tracheal segment (Validyne DP45–32).

### 2.3 Hyoid displacement

**FIGURE 2:**
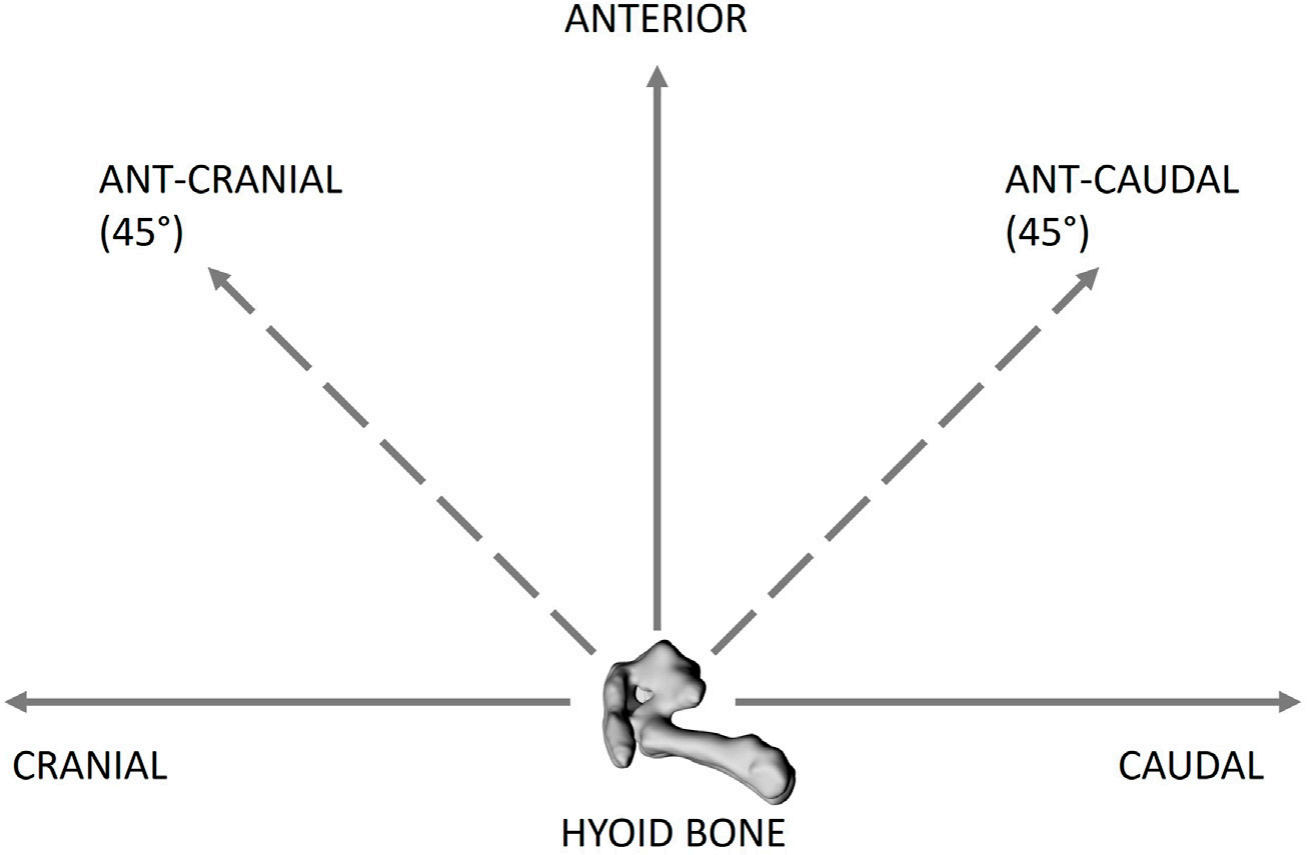
Schematic of the hyoid bone (lateral view) showing the different directions of hyoid bone displacement applied, including cranial, caudal, anterior, anterior-caudal 45° (ant-caudal) and anterior-cranial 45 (ant-cranial). The hyoid bone was displaced from 0 to 5 mm at 1 mm increments in all the directions.

A hyoid repositioning device was developed in-house to allow for quantifiable displacement of the hyoid in set increments and directions, including: anterior, caudal, cranial, anterior-cranial 45° (ant-cranial), and anterior-caudal 45° (ant-caudal) (Figure 2). A miniscrew (RMO^®^ Dual-Top, 2 mm × 8 mm) was inserted centrally into the body of the hyoid for later attachment to the hyoid repositioning device. The hyoid repositioning device consisted of a sliding horizontal platform suspended above the rabbit. A modified digital caliper (at a resolution of 0.01 mm) with a rigid extension and alligator clamp was attached perpendicular to the horizontal platform; it served as the *vertical* caliper. The extension was clamped to the hyoid-inserted miniscrew. The vertical caliper was used to displace the hyoid anteriorly by the required increment and then return it to baseline position. The horizontal sliding platform allowed for cranial-caudal hyoid movements, which were quantified using a second digital caliper, the *horizontal* caliper. Ant-cranial and ant-caudal movements were achieved by increments using both the vertical and horizontal (sliding platform) calipers.

### 2.4 Pclose measurements

**FIGURE 3:**
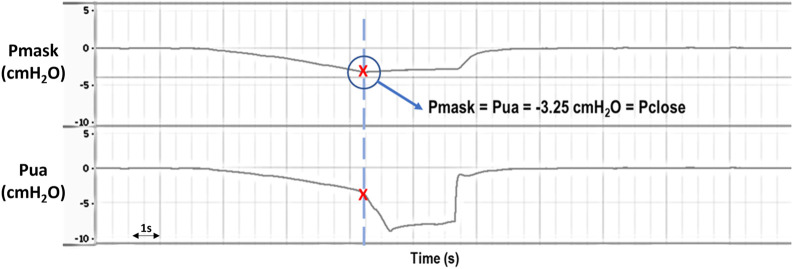
Representative raw data example showing Pmask (mask pressure) and Pua (upper airway pressure) during application of negative intraluminal pressure for Pclose (closing pressure) determination. Initially, Pmask and Pua decrease at the same rate as increasing negative pressure is being applied to the upper airway, indicating an open airway. Eventually there is a deviation in Pua and Pmask, with a sudden drop in Pua while Pmask stabilizes, indicating that the upper airway has closed (i.e., Pclose = −3.25 cmH_2_O in this example). Pmask and Pua were later retuned to 0 cmH_2_O when the system was opened to atmosphere.

Upper airway pressure was reduced progressively using the syringe connected to the upper tracheal segment. Initially, the pressure detected at the level of the mask (Pmask) was the same as the pressure applied at the level of the trachea (Pua), considering that the upper airway was open. Pua and Pmask were monitored carefully until the point of deviation, signifying upper airway closure [see Figure 3]. The minimal pressure value reached by Pmask before diverging from Pua was taken as the closing pressure of the upper airway (Pclose), reflecting upper airway collapsibility; the greater the negative value of Pclose, the less collapsible the airway.

### 2.5 Interventional protocol

Pclose was measured at baseline prior to and following hyoid device attachment. Each measurement was repeated 3 times. The hyoid was then re-positioned within the mid-sagittal plane in sequence along anterior, caudal, cranial, ant-cranial, and ant-caudal directions from baseline (0 mm) to 5 mm in 1 mm increments. Pclose was measured for each direction and increment. Following each Pclose measurement, the system was opened back to atmosphere (0 cmH_2_O) before being closed again, ready for the next measurement. This sequence was repeated three times.

### 2.6 Statistical analysis

For each rabbit, average baseline Pclose values from corresponding repeated measurements were calculated. Baseline values prior to and post hyoid device attachment, and among hyoid directions were compared using two-way ANOVA. To analyze the effects of hyoid displacement, Pclose data were expressed as a change from baseline (ΔPclose). For each rabbit, ΔPclose data from the three repeated measurements for corresponding hyoid direction and increment were averaged to obtain individual rabbit data, which were later used in group analysis. A two-way ANOVA was employed to analyze the effect of the two independent variables (hyoid displacement direction and increment), both individually and their interaction, on ΔPclose (dependent variable). Bonferroni *post hoc* tests, corrected for multiple comparisons, were used to determine differences in ΔPclose among increments and directions. Group data were expressed as mean ± SD. Statistical analyses were performed using SPSS (v20, IBM Corp. Armonk, NY) and statistical significance was set at *p* < 0.05.

## 3 Results

### 3.1 Baseline Pclose

The average baseline Pclose prior to hyoid repositioning device attachment to the hyoid was -3.7 ± 0.7 cmH_2_O, and −3.6 ± 0.9 cmH_2_O following device attachment. The difference in Pclose before and after device attachment was of borderline significance (*p* = 0.045). Average baseline Pclose values prior to anterior, caudal, cranial, ant-caudal and ant-cranial hyoid displacement directions were −3.1 ± 1.2, −3.4 ± 0.9, −3.2 ± 1.0, −3.0 ± 1.3 and −2.7 ± 1.2 cmH_2_O, respectively. Differences between direction baseline values were not statistically significant (*p* > 0.1).

### 3.2 Hyoid displacement directions

**FIGURE 4:**
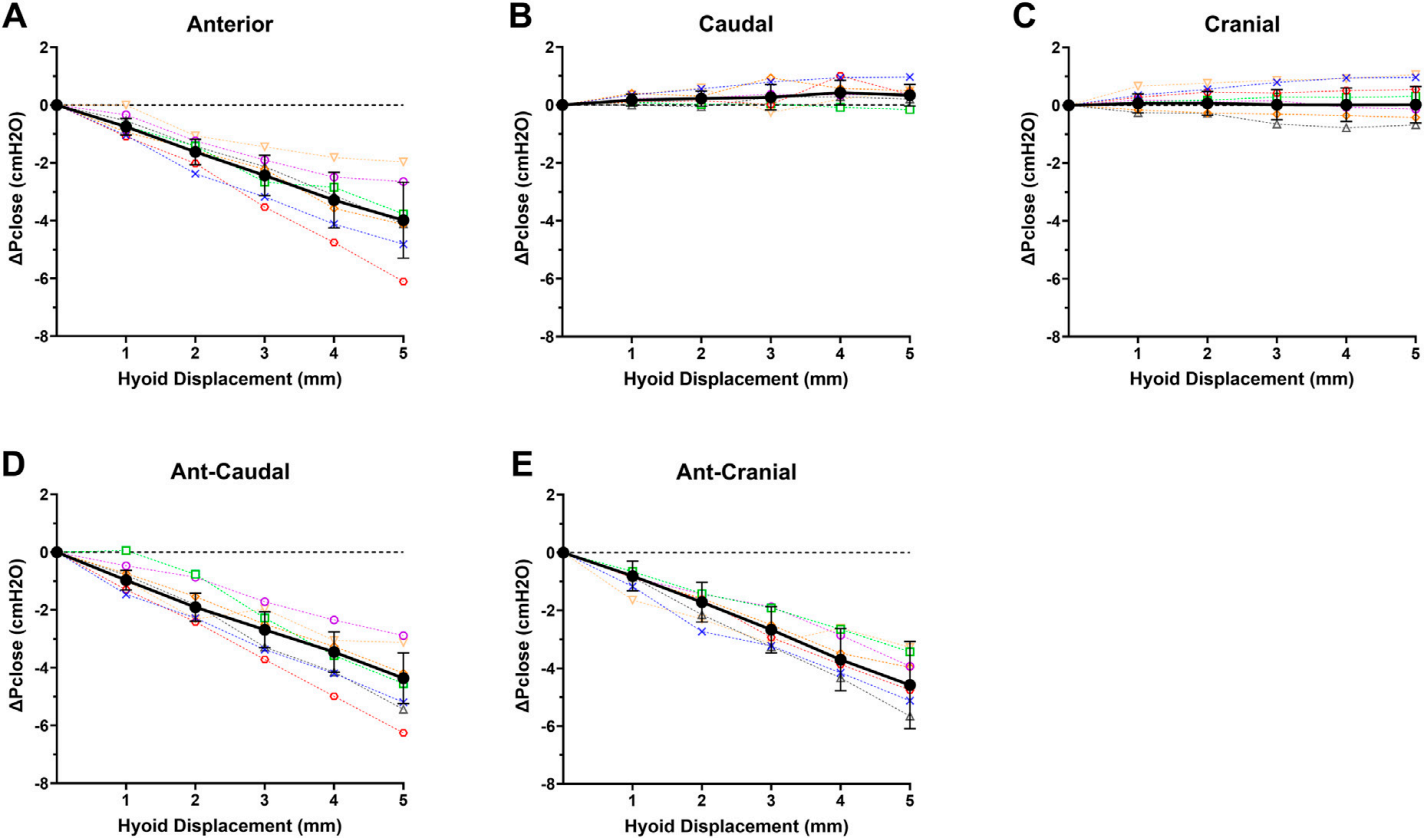
Individual rabbit (*n* = 7; open symbols, dashed interpolated line) and group mean ± SD (closed dark circles with error bars representing standard deviation, solid interpolated line) ΔPclose vs increasing hyoid displacement for each displacement direction: **(A)** anterior, **(B)** caudal, **(C)** cranial, **(D)** ant-caudal, and **(E)** ant-cranial. ΔPclose progressively decreased with hyoid displacement in all anterior based directions **(A,D,E)**, while there was no significant change in ΔPclose for cranial and caudal directions.

ΔPclose decreased progressively with increasing anterior hyoid displacement in all rabbits (Figure 4A). This reduction was significant between all increments (*p* < 0.05), except between 4 and 5 mm (*p* = 0.062) (Figure 4A). At the maximum anterior displacement (5 mm), Pclose dropped to −7.3 ± 1.2 cmH_2_O, and the ΔPclose was −4.0 ± 1.3 cmH_2_O (Figure 4A).

Caudal hyoid displacement did not significantly affect ΔPclose (*p* = 0.352; Figure 4B). Similarly, when the hyoid was moved cranially, overall ΔPclose did not change for the group (*p* = 0.723; Figure 4C).

ΔPclose decreased progressively with increasing ant-caudal and ant-cranial hyoid displacement in all rabbits (Figures 4D,E). In the ant-caudal direction, ΔPclose was significantly different at all levels (*p* < 0.05), except between 2 and 3 mm and between 4 and 5 mm (*p* = 0.078 and *p* = 0.057, respectively). At maximum ant-caudal displacement (5 mm), Pclose reached −7.6 ± 1.4 cmH_2_O, i.e., ΔPclose = −4.6 ± 1.5 cmH_2_O (Figure 4D).

When the hyoid was displaced in the ant-cranial direction, ΔPclose varied significantly at all displacement levels (*p* < 0.05), except between 3 and 4 mm (*p* = 0.149). At maximum ant-cranial displacement (5 mm), Pclose decreased to −7.1 ± 1.1 cmH_2_O, i.e., ΔPclose = −4.4 ± 0.9 cmH_2_O (Figure 4E).

### 3.3 Comparison between directions

The decrease in ΔPclose with anterior hyoid displacement was not significantly different from the ant-caudal (*p* = 0.679) and ant-cranial (*p* = 0.995) displacements. In addition, ΔPclose in ant-caudal and ant-cranial were similar (*p* = 0.847). However, ΔPclose in anterior, ant-caudal and ant-cranial hyoid displacement directions was significantly different from the cranial and caudal measurements for all increments (*p* < 0.03).

## 4 Discussion

This is the first study to comprehensively investigate and quantify the influence of hyoid surgical repositioning in different directions and magnitudes on upper airway collapsibility. The primary outcome of the study was that decreases in upper airway collapsibility associated with hyoid repositioning were dependent on both direction and magnitude of hyoid displacement. More specifically:

1) Anterior hyoid displacement was the main component leading to decreased upper airway collapsibility, with greater increments in displacement progressively leading to a less collapsible upper airway
2) Caudal and cranial hyoid displacements did not alter upper airway collapsibility
3) Ant-caudal and ant-cranial displacements yielded similar upper airway collapsibility outcomes as the absolute anterior displacement direction.

### 4.1 Anterior hyoid displacement

The anterior advancement of the hyoid has long been recognized to potentially benefit the treatment of OSA. Examining the effect of anterior hyoid displacement in human cadavers, Rosenbluth et al. (2012) found that 1 cm of advancement lead to an almost 4-fold improvement in upper airway airflow. Hyoid anterior displacement (magnitude not specified) in intact anesthetized rabbit upper airways resulted in increased inspiratory flow and decreased intraluminal airway pressure swings (Benderro et al., 2018). In a study of the effect of displacement of the hyoid arch on upper airway flow resistance in anaesthetized dogs, Van de Graaff et al. (1984) reported that anterior displacement by an arbitrary amount decreased upper airway resistance by approximately 57% during inspiration. Our findings are consistent with these investigations, revealing an over 2-fold reduction in Pclose with anterior hyoid displacement at the maximum 5 mm increment. The decrease in Pclose was progressive with increment: the greater the hyoid bone anterior displacement, the greater the improvement in upper airway collapsibility.

### 4.2 Caudal and cranial hyoid displacement

Caudal and cranial displacements of the hyoid bone have not been investigated previously without any anterior vector component. We found that caudal and cranial directions did not have any significant effect on upper airway closing pressure at all hyoid displacement increments.

**FIGURE 5:**
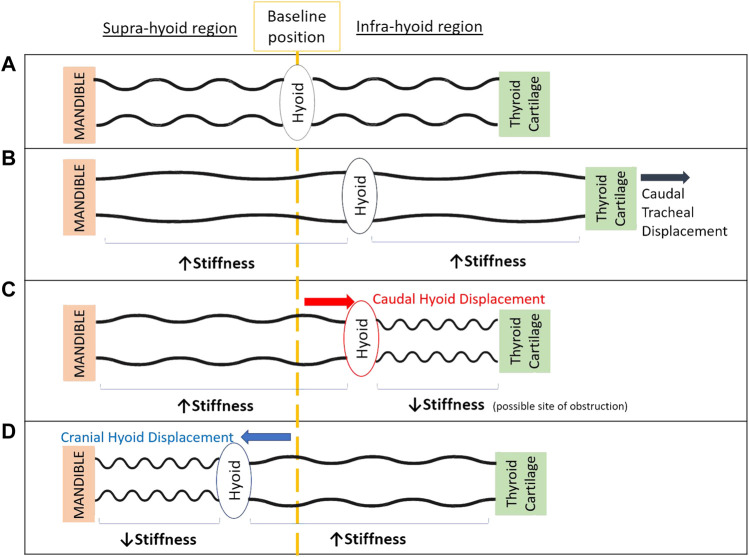
Schematic model of the upper airway demonstrating the potential effect of cranial and caudal hyoid repositioning on supra-hyoid and infra-hyoid upper airway regions to aide in explaining study outcomes. When the hyoid is at its baseline position **(A)**, the upper airway is patent. Displacement of the thyroid cartilage and hyoid caudally with caudal tracheal displacement **(B)**, stretches upper airway soft tissues in both supra- and infra-hyoid regions, hence stiffening the airway’s walls along its length (Amatoury et al., 2014; Amatoury et al., 2016; Tong et al., 2019). Unfolding of the upper airway wall may also occur (Amatoury et al., 2010; Kairaitis et al., 2015). Following direct application of caudal hyoid bone displacement **(C)**, supra-hyoid tissues are stretched, however infra-hyoid tissue (e.g., thyrohyoid, sternohyoid and omohyoid muscles) strains are likely reduced (unlike with tracheal displacement), leading to a “floppier” and more collapsible airway segment in this region. The balance between regions along the length the upper airway of increased and decreased stiffness, overall gives way to no change in upper airway collapsibility. Following cranial displacement of the hyoid bone **(D)**, the soft tissues in the supra-hyoid region (e.g., hyoglossus, styloglossus, palatoglossus) become “floppier” and the airway narrowed, while the infrahyoid region is stretch and stiffened. Similar to caudal hyoid displacement, this likely results in the overall zero change in upper airway collapsibility with cranial hyoid displacement.

A conceptual model to help explain these outcomes is shown in Figure 5. The lack of improvement in upper airway collapsibility with caudal hyoid displacement may initially seem counter intuitive because it generates a stretch of the tissues located in the suprahyoid airway region, making them stiffer, therefore less collapsible (Figure 5B). This effect has been shown to result from caudal hyoid displacement secondary to caudal tracheal displacement in animal and computational modeling studies (Amatoury et al., 2014; Amatoury et al., 2016). However, with direct application of caudal hyoid displacement, while suprahyoid tissues likely become stiffer, the tissues below the hyoid may become softer (“floppier”) as strain is decreased (Figure 5C). We hypothesize that this response of the tissues may change the site of collapse in the upper airway to the infrahyoid region: with increased and decreased stiffness in suprahyoid and infrahyoid upper airway regions, respectively, no change would occur in upper airway collapsibility. Similarly, hyoid displacement in a cranial direction can lead to floppier tissues in the region above the hyoid, thus the upper airway may collapse in the suprahyoid region (Figure 5D). Further study, using imaging, is required to confirm this hypothesis.

### 4.3 Ant-caudal and ant-cranial hyoid displacement

Ant-caudal and ant-cranial hyoid displacement studies have been only directly performed previously in one human cadaver study in which the hyoid was pulled anteriorly and 30° ant-caudally (Rosenbluth et al., 2012). The anterior advancement produced more airway opening and improvement in upper airway airflow compared to the 30° ant-caudal hyoid movement.

In freshly euthanized rabbits, mechanical tension on the hyoid that produced ∼1–2 mm ant-caudal hyoid movement was found to decrease Pclose by −2.6 ± 1.7 cmH_2_O (Roberts et al., 1984). This finding is similar to the Pclose reduction obtained in the current study with 2 and 3 mm ant-caudal (45°) hyoid displacement (i.e., −1.9 ± 0.5 and 2.7 ± 0.6 cmH_2_O, respectively). Another study produced a similar change in the airway when tension was applied to the hyoid to simulate geniohyoid/genioglossus muscle contraction, which moved the hyoid in an ant-cranial direction (Brouillette and Thach, 1979). The amount of hyoid movement was not reported, however the authors predicted that the improvement in airway stability was related to the hyoid movement. In a simulated contraction of the same muscles in infant cadavers through the application of tension to the hyoid, Reed et al. (1985) obtained widening of the pharyngeal airway. Thus, our findings corroborate the notion advanced in other studies that ant-caudal and ant-cranial based hyoid displacements improve upper airway patency and stability.

### 4.4 Comparison of directions

The greatest decreases in Pclose in the current study were with anterior, ant-caudal and ant-cranial displacements of the hyoid bone. The caudal and cranial hyoid displacements did not alter upper airway collapsibility. Thus, it can be deduced that the anterior movement of the hyoid is the primary component leading to improvement in airway collapsibility. Indeed, when combining caudal or cranial displacement components to the anterior movement, the collapsibility of the upper airway remained the same as with anterior alone.

### 4.5 Clinical implications for hyoid suspension surgeries

The available hyoid surgical repositioning techniques have an anterior force vector combined with another vector applied either caudally (suspension to the thyroid cartilage; hyothyroidopexy) or cranially (suspension to the mandible; hyomandibular suspension). Very few studies comparing hyomandibular suspension and hyothyroidopexy have been undertaken. In a systematic review providing a thorough comparison between hyoid surgical techniques and including patients with all OSA severities (Song et al., 2016), hyothyroidopexy was found to reduce the AHI on average by 50.7%, which was more than the reduction with hyomandibular suspension of 38.3%. However, the results for hyothyroidopexy were highly skewed and the surgical technique for hyomandibular suspension used in the reviewed studies was the older more invasive technique initially proposed by Riley et al. (1986) that required considerable myotomy of the hyoid muscles.

A minimally invasive hyomandibular suspension later introduced by Gillespie et al. (2011) involved only minimal muscle cutting and was expected to maximize the effect of hyoid displacement through muscle stretching and further stabilize the airway. This improved technique combined with palatal surgery produced a 62% improvement in AHI and 76.9% success rate among patients with moderate-to-severe OSA (Van Tassel et al., 2021). Nonetheless, others have concluded that hyoid suspension alone was not efficacious for relieving hypopharyngeal obstruction in OSA (Bowden et al., 2005). Thus, the literature remains inconclusive on the efficacy of hyomandibular suspension and hyothyroidopexy surgeries, whether performed alone or as part of a multilevel surgical procedure. Studies are limited to surgical reports that compare patients related outcomes rather than fundamental anatomical post-surgical changes.

Our study suggests that in both surgical procedures, the anterior vector primarily contributes to the improvement observed in OSA patients who underwent hyoid repositioning. A greater anterior displacement of the hyoid is noted with hyomandibular suspension compared to hyothyroidopexy (distance hyoid-mandible > distance hyoid-thyroid cartilage). Therefore, improved outcomes are expected with the mandibular suspension, notwithstanding the fact that several factors such as craniofacial anatomy, muscle rearrangements and tissue concentration also play a role in determining the final outcome of those hyoid surgeries. Also impacting hyoid displacement is hypoglossal nerve simulation and mandibular advancement (Amatoury et al., 2015; ElShebiny et al., 2017; Pae and Harper, 2021), inviting focused research about these associations.

### 4.6 Critique of methods

The rabbit model was ideal for the current study because of its general similarity with the human upper airway structure. Despite obvious anatomical differences, such as craniofacial shape and an overlapping soft palate/epiglottis, the rabbit model was ideal for the current study because of its general similarity with the human upper airway structure. Most importantly, unlike other animals and like the human, the hyoid bone of the rabbit is mobile with no fixed bony attachments (Amatoury et al., 2014; Amatoury et al., 2015). The rabbit model has also been repeatedly used in the fields of respiratory and upper airway physiology with demonstrated applicability of outcomes to humans (Brouillette and Thach, 1980; Olson et al., 1989; Isono et al., 1997; Kirkness et al., 2003a; Kirkness et al., 2003b; Fogel et al., 2005; Kairaitis et al., 2006; Squier et al., 2010; Kairaitis et al., 2012; Amatoury et al., 2014; Amatoury et al., 2015; Amatoury et al., 2016; Schiefer et al., 2020).

As in our previous studies (Amatoury et al., 2014; Amatoury et al., 2015), the upper airway was isolated, thus eliminating the respiratory-related upper airway reflexes induced by pharyngeal pressure/flow changes, similar to the normal sleeping condition. This initial step was required to understand the effects of hyoid repositioning on upper airway patency. The presence of muscle activity may alter reported outcomes. This could be quantified with EMG along with airflow and upper airway pressures in an intact upper airway (Song and Pae, 2001; Kairaitis et al., 2012) to determine the influence of specific muscles on hyoid repositioning and the associated upper airway responses, including upper airway resistance.

Pclose of the upper airway has been used in previous studies as a reproducible measure of upper airway collapsibility in animals and humans (Brouillette and Thach, 1979; Abu-Osba et al., 1981; Roberts et al., 1984; Kirkness et al., 2003a; Isono et al., 2003; Isono et al., 2004). The methodology used to measure Pclose in the present study is based on that previously described in dogs (Crawford et al., 1996) and rabbits (Olson et al., 1989; Kirkness et al., 2003a; Kairaitis et al., 2007; Lam et al., 2008). Pclose measured in rabbits under light and deep pentobarbital anesthesia revealed values of +1.1 ± 1.9 cmH_2_O and −4.9 ± 1.4 cmH_2_O, respectively (Abu-Osba et al., 1981). The later deep anesthesia Pclose value is in concordance with our results. These findings further support the fact that rabbits in our study were deeply anaesthetized, and that the upper airway was mostly passive. Similar to previous studies (Olson et al., 1989; Kirkness et al., 2003a; Kairaitis et al., 2007; Lam et al., 2008), Pclose was not measured during a specific respiratory phase in the current study. Considering that the upper airway was isolated with no upper airway airflow and muscle activity, the effect of respiratory phase on Pclose was likely minimal.

The position of the mandible was not fixed in the current study. The mask provided some restriction to potential mandibular movements with hyoid displacement. These movements were unlikely to have changed the observed pattern of responses. Nevertheless, the variability of mandibular movements should be explored in future studies.

Hyoid displacement directions were not applied randomly, but in the same sequence for each animal, i.e. anterior, caudal, cranial, ant-caudal, ant-cranial. Randomization might have avoided a possible order-effect, although the results were consistent regarding the benefit of the anterior direction. Further investigation should determine the potential impact of operational sequence on study outcomes.

## 5 Conclusion

Incremental increase of hyoid displacement in anterior, ant-caudal and ant-cranial directions were associated with a progressive reduction in upper airway closing pressure and hence less collapsible upper airway. Surgical re-positioning of the hyoid in caudal or cranial directions had no effect on upper airway collapsibility. The findings suggest that changes in upper airway collapsibility are dependent on both direction and magnitude of hyoid repositioning, and that the anterior component has the greatest impact on reducing upper airway collapsibility. The results may have implications for guiding and improving the outcomes of surgical hyoid repositioning interventions for the treatment of OSA.

## Data Availability

The raw data supporting the conclusion of this article will be made available by the authors, without undue reservation.
